# Intestinal mucosal tolerance and impact of gut microbiota to mucosal tolerance

**DOI:** 10.3389/fmicb.2014.00781

**Published:** 2015-01-13

**Authors:** Dimitry A. Chistiakov, Yuri V. Bobryshev, Emil Kozarov, Igor A. Sobenin, Alexander N. Orekhov

**Affiliations:** ^1^Department of Medical Nanobiotechnology, Pirogov Russian State Medical University, Moscow, Russia; ^2^The Mount Sinai Community Clinical Oncology Program, Mount Sinai Comprehensive Cancer Center, Mount Sinai Medical Center, Miami Beach, FL, USA; ^3^Research Center for Children’s Health, Moscow, Russia; ^4^Institute of General Pathology and Pathophysiology, Russian Academy of Sciences, Moscow, Russia; ^5^Faculty of Medicine, School of Medical Sciences, University of New South Wales, Sydney, NSW, Australia; ^6^School of Medicine, University of Western Sydney, Campbelltown, NSW, Australia; ^7^Department of Oral and Diagnostic Sciences, Columbia University, New York, NY, USA; ^8^Laboratory of Medical Genetics, Russian Cardiology Research and Production Complex, Moscow, Russia

**Keywords:** intestinal microbiota, immune system, microflora, tolerance, harmful antigens

## Abstract

The mucosal barriers are very sensitive to pathogenic infection, thereby assuming the capacity of the mucosal immune system to induce protective immunity to harmful antigens and tolerance against harmless substances. This review provides current information about mechanisms of induction of mucosal tolerance and about impact of gut microbiota to mucosal tolerance.

## INTRODUCTION

The human intestine harbors a whole microbial ecosystem containing over 100 trillion microorganisms that collectively have a total genome (the microbiome) consisting of 100-fold more genes than the human genome (Tsai and Coyle, 2009). The gut microbiota lives in tight symbiosis and homeostasis with the host and plays an essential role in harvesting energy, minerals, and bioactive compounds from the food. The intestinal microbiota exists in reciprocal balance with the gut-associated lymphoid tissue (GALT), the largest immune system in the body. Gut microorganisms are the major source of natural antigens that continuously stimulate the GALT and induce mucosal immune tolerance (e.g., local or systemic immune unresponsiveness) to innocuous antigens such as food proteins and molecular components of commensal bacteria (Pabst and Mowat, 2012). In addition, the GALT is necessary for preventing acute proinflammatory immune responses against the microbiota resulting in inflammatory bowel diseases or against food protein causing food allergy and celiac disease. The mucosal barriers are very sensitive to pathogenic infection thereby assuming the capacity of the mucosal immune system to induce protective immunity to harmful antigens and tolerance against harmless materials (Weiner et al., 2011).

Oral tolerance is a phenomenon of suppressing immune responses in the gut and systemic immune system by orally administered antigens. Tolerance to intestinal bacteria and tolerance to food proteins differs by its effects on the immune system. Tolerance to food protein induced through the small intestine influences both local and systemic immune responses, while tolerance to gut bacteria in the colon does not attenuate systemic responses (Pabst and Mowat, 2012). Oral tolerance was extensively studied in rodents using many different antigens such as purified proteins, cellular antigens, and small haptens. The phenomenon of oral tolerance was also described in humans (Kraus et al., 2004; Kapp et al., 2010). The effects of oral tolerance on the immune system were usually evaluated as a suppression of cytokine production and T-cell proliferation and decrease in serum titers of circulating antibodies. The ability of oral tolerance to inhibit autoimmune and inflammatory diseases was demonstrated in rodent experimental models of type 1 diabetes, encephalomyelitis, arthritis, myasthenia, thyroiditis, and other pathology (Faria and Weiner, 2006). Thus, oral tolerance is able to diminish a broad spectrum of immune responses and hence could play a key role in maintaining peripheral immune homeostasis.

## MECHANISMS OF INDUCTION OF MUCOSAL TOLERANCE

### INTESTINAL INTAKE OF TOLEROGENIC ANTIGEN

The intestinal immune system is composed by several essential components such as GALT [Peyer’s patches (PPs) and isolated lymphoid follicles (ILFs)] and gut-draining mesenteric lymph nodes (mLNs), which primarily contribute to the induction of mucosal tolerance through recognition of colon-derived bacterial and viral antigens (Brandtzaeg et al., 2008). In the epithelium of PPs and ILFs, specialized epithelial cells called as microfold cells (M cells) are involved in the permanent transfer of colon material from the gut lumen into the GALT (Miller et al., 2007). The intestinal antigen is then passed from M cells on to dendritic cells (DCs) that reside either below the epithelium or in a “pocket” created at the basolateral surface of the M cell. Antigen uptake by DCs in the lamina propria (LP) underlying regular villus epithelium was shown to be critical for inducing tolerance to soluble antigens in the small intestine (Chirdo et al., 2005).

There are several possible mechanisms of delivery of intestinal antigens from the lumen to DCs. Low-molecular antigens such as haptens and peptides could diffuse through the intestinal epithelium *via* pores in inter-epithelial tight junctions (Hossain and Hirata, 2008). High-molecular complexes can be taken across enterocytes by transcytosis or through exosome-mediated pathway associated with major histocompatibility complex (MHC) class II-dependent recognition and antigen processing (Menard et al., 2010). DCs were shown to efficiently uptake exosomes. The exosomes containing MHC class II associated with gut antigenic peptides were able to induce tolerance in recipient mice after isolation from serum of antigen-fed animals (Karlsson et al., 2001).

In fact, the antigen nature drives its path of uptake. Particulate material and microbiota are mainly delivered into the GALT by transcytosis throught M cells while soluble antigens induce oral tolerance after DC-mediated intake mostly in the LP and then in the GALT.

### DISSEMINATION OF GUT ANTIGEN WITHIN THE BODY

Orally administered antigens are likely to disseminate across the body through the circulation. For example, food protein can be found in the blood of humans soon after meal intake (Husby et al., 1985). The antigen entry to the bloodstream occurs not simply but is accompanied with detectable changes in the mucosal immune system including activation of C-type lectin (marker CD69) expression and T cells in mLNs and peripheral LNs (Smith et al., 2002). Furthermore, since serum-derived exosomes from antigen-fed animals could induce tolerance in naïve recipient animals, this phenomenon indicates the presence of tolerogenic material (Karlsson et al., 2001, 2010). Indeed, it is important to know where in the body the gut antigen induces oral tolerance.

The administration of an antigen into the portal vein induces tolerance that is specific to the antigen (Thomson and Knolle, 2010) whereas disruption of the intrahepatic blood flow by the portocaval shunt prevents oral tolerance induction (Yang et al., 1994). These findings support the liver as a likely tolerogenic site for gut antigen. Furthermore, the liver is anatomically located as the endpoint of the portal vein delivering blood directly from the intestine.

The liver is enriched with specialized antigen-presenting cells (APCs) that could be primarily involved in the tolerance induction. Kupffer cells and conventional hepatic DCs belong to professional APCs challenging immune responses against gut antigens in favor to inducing and maintaining tolerance (Thomson and Knolle, 2010). In addition, hepatic sinusoidal endothelial cells are able to collect circulating antigens and act as APCs in inducing tolerance (Limmer et al., 2005; Holz et al., 2010). In the liver, plasmacytoid DCs especially contribute to the induction of systemic tolerance to orally administered antigens by down-regulating and initiating anergy in antigen-specific CD4^+^ and CD8^+^ T cells (Goubier et al., 2008; Dubois et al., 2009).

In the spleen and peripheral LNs that are located beyond the liver, resident DCs could trigger local and systemic tolerance to the gut-derived antigen even the absence of costimulation through initiating anergy in effector T cells or inducing regulatory T cells (Tregs; Yamazaki et al., 2008) but with less efficiency than GALT-associated DCs do (Hashiguchi et al., 2011). However, it is likely that intestinal DCs play a key role in inducing systemic tolerance.

### GALT-ASSOCIATED DCs PLAY A CRUCIAL ROLE IN INDUCING ORAL TOLERANCE

Gut antigen-induced CD103^+^ DCs migrating from the LP to mLNs are responsible for major delivery and recognition of colon-derived antigens in the GALT (Pabst et al., 2007). The travel of DCs from LPs to mLNs is dependent on C-C chemokine receptor (CCR) 7, a chemokine receptor (Forster et al., 2008). The lack of all LNs and PP in lymphotoxin α-deficient mice leads to the loss of oral tolerance that could be restored by specifically induced mLN formation (Spahn et al., 2002). Similarly, surgical deletion of mLNs in mice abolishes the induction of oral tolerance (Worbs et al., 2006). These findings suggest that the intestine immune system and especially mLNs have a primary role in the induction of oral tolerance.

Gut-associated lymphoid tissue-associated DCs that express on their surface integrin chain-αE (CD103) never reach the circulation beyond mLNs (Milling et al., 2010). In LPs, intestinal CD103^+^ DCs recognize gut antigens and possess tolerogenic and immunoregulatory properties stimulating expression of homing molecules CCR7 and integrin-α_IV_β_7_ on T cells resided in the mLNs and inducing Forkhead box protein 3 (FoxP3)-positive Tregs (Johansson-Lindbom et al., 2005; Sun et al., 2007; Jaensson et al., 2008; Worthington et al., 2011). Gut-derived vitamin A and other retinoids were shown to modulate homing-inducing and tolerogenic properties of CD103^+^cells by inducing synthesis of homing molecules CCR9 and CCR4 (Iwata et al., 2004; Jaensson-Gyllenbäck et al., 2011). Retinoic acid was reported to down-regulate experimentally induced intestinal inflammation (ileitis) by restoring the balance between proinflammatory Th17 cells and inducible CD4^+^FoxP3^+^ Tregs (iTregs) in the mouse (Collins et al., 2011). Furthermore, retinoic acid produced by CD103^+^DCs cooperates with transforming growth factor-β (TGF-β) in generation of iTregs from naive CD4^+^T cells (Sun et al., 2007). The integrin-α_IV_β_7_ was found to be significantly up-regulated on the surface of CD103^+^DCs and activates latent TGF-β by releasing it from the complex with latent TGF-β-binding protein (LTBP) thereby mediating TGF-β-dependent induction of Tregs (Païdassi et al., 2011; Worthington et al., 2011). Intestinal inflammation impairs function of GALT-associated CD103^+^ cells and abolishes their tolerogenic activities (Laffont et al., 2010).

CD103^+^ DCs were observed to cooperate with other cell types presented in the intestinal mucosa to induce gut tropism in T cells and oral tolerance. In mLNs, non-hematopoietic stromal cells are essential for full display of ability of GALT-associated DCs to induce gut-homing T cells (Hammerschmidt et al., 2008). Notably, stromal cells taken from peripheral LNs failed to initiate gut tropism in T cells. Furthermore, mLN-derived stromal cells produce high levels of retinoic acid-metabolizing enzymes that are essential for retinoic acid-mediated induction of synthesis of homing molecules (Molenaar et al., 2009). Similarly, stromal cells from mLNs but not from skin-draining LNs support the generation of Foxp3^+^ Tregs (Cording et al., 2014). Thus, gut-specific environment and synergistic interactions of mLN-derived stromal and DCs play a crucial role in induction of intestinal T cell homing and gut-associated Tregs.

Recently, a new population of intestinal CD103^–^ DCs was identified (Cerovic et al., 2013). Like CD103^+^ DCs, these cells are responsive to Flt3 (FMS-like tyrosine kinase 3), a regulatory factor crucial for the hematopoietic commitment and functional and phenotypic maintenance of DCs, and prime a gut-homing phenotype to naive T cells in the mLNs. CD103^–^CD11b^+^ CX_3_CR1^int^ lymph DC subset induce the differentiation of proinflammatory interferon (IFN)-γ and interleukin (IL-17)-17-producing effector T cells (Cerovic et al., 2013). Administration of Flt3 ligand resulted in inducing CD103 expression in CD103^–^ DCs and converting these cells from proinflammatory to tolerogenic CD103^+^ DCs that contributed to generation of CD4^+^FoxP3^+^ Tregs and attenuation of experimental Crohn’s-like ileitis (Collins et al., 2012). Therefore, local microenvironment and proinflammatory/anti-inflammatory stimuli could greatly influence the phenotype and function of GALT-associated DCs.

### MECHANISMS OF ORAL TOLERANCE INDUCTION

Oral tolerance utilizes mechanisms similar with those of the peripheral immune tolerance including active impact of Tregs, clonal deletion, and clonal anergy of T cells. Antigen doses were shown to influence the choice of tolerogenic mechanisms and numbers of iTregs (Weiner et al., 2011). In exposure to a single high dose of antigen, clonal deletion or anergy are preferential tolerogenic mechanisms whereas numerous low antigen doses induce T cell anergy (Chen et al., 1996). In oral tolerance, the highest counts of Foxp3^+^ Tregs were induced after exposure to the high doses of an antigen (Siewert et al., 2008).

Inducible Tregs generated with contribution of intestinal CD103+ DCs and mLN-specific stromal cells appears to play a key role in induction and maintenance of oral tolerance. Several subsets of Tregs such as CD4^+^FoxP3^+^ iTregs, IL-10-producing regulatory type 1 cells (Tr1), and TGF-β-producing Th3 Tregs were shown to be involved in oral tolerance (Pabst and Mowat, 2012). Adoptive transfer of CD4^+^CD25^+^FoxP3^+^ Tregs from tolerogenic animals could induce oral tolerance in naïve mice whereas depletion of FoxP3^+^Tregs abolishes tolerance (Dubois et al., 2003). FoxP3^+^ and FoxP3^–^ IL-10-producing Tregs were found in the gut mucosa (Maynard et al., 2007). Interestingly, *all-trans* retinoic acid was shown to have reciprocal effects on induction of Foxp3 and IL-10 in developing CD4^+^ Tregs. By enhancing TGF-β-dependent Foxp3 induction, *all-trans* retinoic acid inhibits TGF-β-dependent induction of IL-10-producing Tregs. Toll-like receptor (TLR)-9-mediated suppression of *all-trans* retinoic acid production by GALT-associated DCs alternately induces preferential expression of IL-10 in Tregs (Maynard et al., 2009).

Among subsets of Tregs, the role of FoxP3^+^ Tregs [e.g., natural CD4^+^CD25^+^FoxP3^+^ Tregs (nTregs) and iTregs] in oral tolerance is the best studied (Curotto de Lafaille et al., 2009). nTregs are selected in the thymus and are responsible for driving central tolerance whereas iTregs are induced in the periphery and are accordingly involved in the regulation of peripheral tolerance. In changing microenvironmental conditions, nTregs maintain a stable phenotype although their function could be impaired (Rubtsov et al., 2010). In contrast, iTregs have a plasticity to differentiate to other helper T cell types under inflammatory stimuli (Koenecke et al., 2009). However, in steady-state non-inflammatory conditions, FoxP3^+^ Tregs can induce and maintain oral tolerance for a long time. Interestingly, in a mouse model, nTregs were unable to induce tolerance to oral ovalbumin while iTregs did suggesting for the obligatory need in peripheral conversion of naïve CD4^+^ T cells to iTregs (Curotto de Lafaille et al., 2008).

Indeed, mLNs provide an essential gut-specific microenvironment for selective differentiation of iTregs (Hadis et al., 2011).

### MAINTENANCE OF ORAL TOLERANCE

Assuming that the mucosal immune system every day should respond to new intestinal antigens and oral tolerance to a specific antigen could be maintained for several months, iTregs are likely to be continuously generated in the GALT (Strobel and Ferguson, 1987). Induction and maintenance of oral tolerance is a multi-stage process involving lymphoid and mucosal tissues (Hadis et al., 2011). After induction in mLNs, iTregs should keep gut-specific homing for long-term supporting oral tolerance. Expression of homing molecules such as integrin-β_7_ and its ligand MadCAM-1 (mucosal vascular addressin cell adhesion molecule 1) is essential for supporting GALT-associated homing of newly generated iTregs (Wagner et al., 1996; Gorfu et al., 2009). Depletion of these two molecules results in greatly diminished and impaired oral tolerance that could be restored by adoptive transfer of integrin-β7-positive T cells (Hadis et al., 2011).

CCR9 was shown to be crucial for the maintenance of GALT-associated homing of T cells since CCR9-null mice have marked defects in oral tolerance (Cassani et al., 2011). CCR9 and integrin-α_IV_β_7_ are both required for small intestine-specific homing of immune cells (Svensson et al., 2002; Stenstad et al., 2007). Retinoic acid is required for induction of CCR9 expression in T cells. Activation of the retinoic acid receptor (RAR) and retinoid X receptor (RXR) results in expression of high levels of CCR9, integrin-αIVβ7, and FoxP3 essential for differentiation of naïve T cells to iTregs and their homing in the small-intestine-associated GALT (Takeuchi et al., 2010).

In the small intestine LP, iTregs induced in mLNs are subjected to secondary expansion that is mediated by the chemokine receptor CX_3_CR. This receptor is also crucial for induction of oral tolerance because CX_3_CR-null mice lack oral tolerance. In the LP of CX_3_CR-null mice, myeloid cells secrete less IL-10, and its production could be rescued by adoptive transfer of wild-type macrophages that express IL-10 (Hadis et al., 2011). Due to decreased production of IL-10, generation of iTregs is suppressed in CX_3_CR-null mice, an event, which in turn abrogates the induction of oral tolerance (Murai et al., 2009). IL-10-producing intestinal mucosa-resident macrophages could therefore contribute to maintenance of iTregs and iTregs-dependent oral tolerance (Gonnella et al., 1998).

Indeed, oral tolerance associated with the small intestine is induced by the cooperative action of mLN-derived CD103^+^ DCs and stromal cells that produce retinoic acid required for imprinting of gut-homing molecules on specific T cells. Certain T cells differentiate to iTregs that leave the mLN and home to the small intestine where they undergo secondary expansion activated by IL-10-secreting CX_3_CR^high^ myeloid cells and resident macrophages. Some secondarily expanded Tregs can possibly leave the GALT and enter the circulation *via* lymphatic vessels or directly to the bloodstream thereby contributing to expanding tolerance to orally administered antigen from local (e.g., gut-associated) to systemic tolerance. The mechanism of establishment of systemic oral tolerance could be similar with the trafficking of CD4^+^Foxp3^+^ Tregs from skin draining LNs to the skin where they display inhibitory effects and then come back to the LNs (Tomura et al., 2010).

Regular stimulation by gut antigen also contributes to maintaining Tregs in the gut. The T-cell receptor (TCR) repertoire of Tregs from the small intestinal LP is highly overlapping with the TCR repertoire of Tregs from gut-draining mLNs (Föhse et al., 2011). A substantial number of GALT-associated iTregs is likely to arise after induction by colonic microbiota-derived antigen (Lathrop et al., 2011). Thymic nTregs were shown to have the TCR repertoire that is skewed toward self-antigens while the TCR repertoire of iTregs is biased toward non-self-antigens (Hsieh et al., 2006). However, the TCR repertoire of thymus-derived Tregs in colon-associated lymphoid and non-lymphoid tissues is heavily influenced by the composition of the microbiota suggesting that nTregs are involved in mucosal tolerance to commensal microorganisms (Cebula et al., 2013).

**FIGURE 1:**
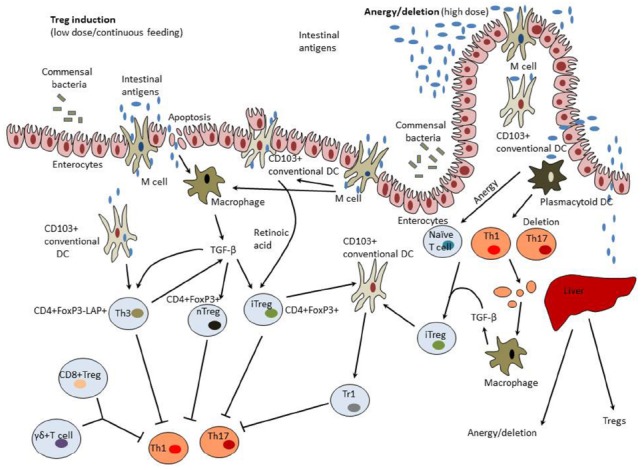
**Mechanisms of induction of mucosal tolerance.** Intestinal antigens could pass from the gut to the GALT through M cells, be collected by DCs, and taken up by enterocytes. GALT-associated DCs have a unique capacity to direct differentiation of regulatory T cells (Tregs) from forkhead box protein 3 (FoxP3)^–^ T cells. These properties of tolerogenic dendritic cells (DCs) are modulated by commensal colon microbiota, transforming growth factor-β (TGF-β), and interleukin (IL-10) produced by enterocytes. Tolerogenic DCs secrete retinoic acid that is synthesized from dietary vitamin A and is essential for formation of inducible Tregs (iTregs). CD11b monocytes also contribute to the induction of Tregs. Treg induction occurs in mesenteric lymph nodes and involves C-C motif receptors (CCR)-7 and -9. Low doses of intestinal antigen lead to the induction of Tregs while high antigen doses result in tolerance induction preferentially through the mechanisms of anergy and deletion. Macrophages activated by clearance of apoptotic T cells and enterocytes become to produce TGF-β and therefore could possess tolerogenic properties. In the liver, high antigen doses could be taken up by tolerogenic plasmacytoid DCs that induce anergy/deletion and Tregs. A variety of Tregs could be induced including CD4^+^CD25^+^Foxp3^+^ natural Tregs (nTregs), CD4^+^CD25^+^Foxp3^+^ iTregs, latency-associated peptide (LAP)^+^ Tregs (Th3 cells), CD8^+^ Tregs, and γδT cells.

Mechanisms of induction of mucosal tolerance are summarized in Figure 1.

## IMPACT OF GUT MICROBIOTA TO MUCOSAL TOLERANCE

### THE STABLE GUT MICROBIOTA IS BENEFICIAL FOR HUMAN HEALTH

The regular intake of beneficial microorganisms (probiotics) is believed to confer health advances on the host. The probiotics such as lactobacilli and bifidobacteria exhibit a range of beneficial effects on the host health including competition with pathogenic microbes for nutrients, supply with vitamin K, inactivation of xenobiotics, stimulating colon peristalsis, digestion of indigestible food fibers, and modulation of the host’s immune system (Hardy et al., 2013). In addition, non-pathogenic commensal bacteria such as *Escherichia coli* were shown to inhibit growth and expansion of pathogens through secretion of antimicrobial peptides (bacteriocin, colicin M, microsin S; Kamenšek and Žgur-Bertok, 2013). Furthermore, several probiotics could cooperate (e.g., form synbiotics) in order to selectively promote the growth of one or more beneficial probiotic species (van Zanten et al., 2012).

The human body plays host to communities of beneficial commensal microorganisms (gut microbiota) that mediate key physiological processes in exchange for nutrients and a sheltered habitat in which they are able to reproduce. In the human intestinal commensal microbiota, the Firmicutes and the Bacteroidetes are the two prevalent phylogenetic types (Eckburg et al., 2005). The stomach contains low numbers of commensal bacteria with predominating species of *Lactobacillus*, *Streptococcus*, and *Helicobacter pylori*. In the small intestine, *Streptococcus* and *Lactobacillus* species are predominant. In the large intestine and distal gut, the *Bacteroides*, *Clostridium*, *Fusobacterium*, and *Bifidobacterium* are prevalent (Eckburg et al., 2005). Indeed, since strain and density range of commensal bacteria greatly vary along the gastrointestinal tract, antimicrobial peptide production and effects of microbiota to the host’s immune system also markedly vary from one location to another in the gut. In pathogenic conditions such as inflammatory bowel disease, these beneficial stable microbiomes are subjected to dramatic changes associated with decreased microbial diversity and unfavorable shift toward increased numbers of gram-negative bacteria (Qin et al., 2010).

The colonization of the newborn intestine is the important stage in the development of future stable gut microbiota. Lower numbers of initial colonizing bacteria, in particular bifidobacteria, may impede establishment of a stable gut microbiota during a critical period of immune education and development. Human milk have was found to provide early immune education and passive immunity through the cooperative action of various bioactive molecules and cells including immunoglobulins, lysozyme, lactoferrin (Grzelak et al., 2014) as well as being a source of commensals such as *L. acidophilus*, *L. gasseri*, *Bifidobacterium bifidum*, and *Bifidobacterium breve* (Martín et al., 2003).

The generation of germ-free mice provided a good option for evaluating the role of the microbiota composition on the immune system. The lack of microbial stimulation leads to maturation defects in lymphoid organs such as PPs and spleen, abnormalities in numbers of immune cells, and altered cytokine expression (Pollard and Sharon, 1970; Bartizal et al., 1984; Williams et al., 2006). Some gut bacteria were shown to play a significant role in the development of GALT. *Bacteroides fragilis* (*B. fragilis*) and *Bacillus subtilis* could stimulate transcytosis in M cells through induction of stress responses to secretion of bacterial protein YqxM essential for sporulation and biofilm formation (Rhee et al., 2004; Chu et al., 2006).

The effects of imbalanced microbiota are not restricted to gastrointestinal abnormalities but could have systemic impact on immunity especially in allergic disorders (Russell et al., 2013) and autoimmune diseases such as multiple sclerosis (Ochoa-Repáraz et al., 2010) and type 1 diabetes (King and Sarvetnick, 2011). Such findings further support the “hygiene hypothesis” suggesting that the absence of immune challenges, result in the insufficient maturation of the immune system and predispose to certain allergic and autoimmune disorders such as celiac disease, asthma, inflammatory bowel disease, type 1 diabetes, etc. (Strachan, 1989).

### INTESTINAL MICROBIOTA AND IMMUNE HOMEOSTASIS

The intestinal microbiota is a crucial component of the immunologic milieu that creates the substrate for oral tolerance (Strober, 2009). The recognition of intestinal bacteria triggers the choice of the subsequent GALT-mediated immune response that should be positive (e.g., stimulatory) in case of pathogens or negative (e.g., tolerogenic) in case of commensal microbes. The host innate immunity of host organism is primarily responsible for recognition of pathogens.

Toll-like receptors and other pathogen-sensing molecules that are highly expressed by enterocytes and mucosal APCs (DCs and macrophages) respond to pathogen-associated molecular patterns (PAMPs; Chu and Mazmanian, 2013). The recognition of pathogens results in induction of antiviral or proinflammatory response against infection.

In the gut epithelium, expression of TLRs is down-regulated on the apical membrane compared to the basolateral side. Low expression of TLR2 and TLR4 is observed on the apical membrane. These receptors prime tolerance to cell wall constituents of commensal bacteria such as lipopolysaccharides (LPS) and peptidoglycans (Fukase et al., 2003; Abreu, 2010). Normally, *E. coli* flagellin does not induce any TLR5-mediated inflammatory response. However, basolateral activation of TLR5 by flagellin derived from pathogenic bacteria such as *Salmonella* results in induction of the acute intestinal inflammation associated with transfer of pathogenic flagellin through gut epithelial cells (Gewirtz et al., 2001). Therefore, when stable and normal microbiota is present in the gut, intestinal epithelial cells are irresponsive to flagellin while flagellin-dependent stimulation of the basolateral epithelial surface is recognized as pathogenic and stimulates TLR5-mediated proinflammatory response (Maaser et al., 2004).

Gram-negative commensal bacteria are major LPS producers in the gut. The epithelial alkaline phosphatase dephosphorylates bacterial LPS that become tolerogenic due to inability to stimulate TLR9 (Lee et al., 2007). In the intestinal epithelium, alkaline phosphatase is concentrated closely to the apical membrane thereby contributing to induction of either inflammatory or immunosuppressive tolerogenic response (Lee et al., 2006). Indeed, bacterial PAMPs are involved not only in the induction of activatory immune responses but also in induction of intestinal tolerance.

The GALT and intestinal epithelium can explore several options to down-regulate TLR-dependent immune stimulation including decrease of TLR expression, release of soluble immune receptors such as soluble TLR2, TLR4, and ST2 (Schmitz et al., 2005), and up-regulation of intracellular inhibitors of TLR signaling including MyD88s (a splice variant of myeloid differentiation factor 88), Toll-interacting protein (Tollip), TNF-related apoptosis-inducing ligand receptor (TRAIL-R), selective androgen receptor modulator (SARM), and others (Shibolet and Podolsky, 2007). Decoy receptors such as ST2 ligand and single Ig IL-1-related receptor (SIGIRR) also could contribute to colonic epithelial homeostasis by inhibiting TLR-induced gut inflammation (Xiao et al., 2007).

A growing number of evidence shows that intestinal commensal microbiota modulate Treg-mediated responses essential for establishing effective protection against pathogens and prevention of autoimmunity, food allergy, gut hypersensitivity to gut-derived antigens, and other unpleasant immunopathologic conditions. Germ-free mice colonized with commensal *Clostridium* species taken from the normal human microbiota developed IL-10-producing CD4^+^FoxP3^+^ Tregs (Atarashi et al., 2011, 2013). The community of 17 strains of *Clostridia* provided bacterial antigens and a TGF-β-rich environment to support induction, proliferation, and expansion of Tregs. Colonization of germ-free animals with human commensal *B. fragilis* induced development of FoxP3^+^ Tregs associated with production of anti-inflammatory cytokines (Round and Mazmanian, 2010). *B. fragilis* was found to secrete polysaccharide A. It has also been shown that *B. fragilis* mediates the conversion of CD4^+^ T cells into Foxp3^+^ Treg cells that produce IL-10 (Round and Mazmanian, 2010). Furthermore, bacterial polysaccharide A was not only able to prevent but also heal experimental colitis in animals suggesting for the involvement of gut commensal microbe *B. fragilis* to mucosal tolerance (Round and Mazmanian, 2010). In addition to live microbiota, LPS derived from the intestinal commensal bacteria could also modulate the mucosal immunity. In germ-free mice, LPS-rich sterile diet led to GALT-associated proliferation and expansion of CD4^+^ T cells and notably to expansion of Foxp3^+^ Tregs in mLNs. Both intestinal microbiota and LPS-rich diet were able to increase production of proinflammatory IL-12 and decrease production of IL-4 essential for differentiation of naïve T cells to pro-inflammatory Th2 cells (Hrncir et al., 2008).

Long-term treatment with antibiotics were shown to deplete normal gut microbiota causing systemic decrease in proliferation of CD4^+^ T cells while Foxp3^+^ Treg proliferation was only locally impaired in the GALT (Cording et al., 2013). In line with this, MyD88-null mice as well as animals deficient for various TLRs showed normal or even increased proliferation of conventional CD4^+^ T cells and Foxp3^+^ Tregs (Atarashi et al., 2011). Indeed, these findings suggest that TLR-mediated recognition of colon-derived bacterial components is not a major mechanism of induction of T cell homeostasis driven by commensal microbiota (Cording et al., 2013). Except for TLRs, other molecular sensors of microbe-derived antigens exist. Those include RIG-I-like receptors (RLPs), NOD-like receptors (NLRs), and DNA-sensing cytosolic receptors (Kumar et al., 2011; Jin and Flavell, 2013). Thus, other, TLR-independent PAMP-sensing mechanisms or metabolic influences could also be involved in the colonic microbiota-dependent control of T cell proliferation.

Animal models of experimentally induced autoimmune disorders suggest the link between the microbial inhabitants of the gastrointestinal tract and autoimmunity. Non-obese diabetic (NOD) mice, a model for type 1 diabetes, which lacks MyD88, an essential signaling component of innate immunity linking microbe-sensing immune receptors with immune signaling cascades is resistant to type 1 diabetes. However, germ-free MyD88-null NOD mice develop severe diabetes while colonization of germ-free MyD88-null NOD mice with microbiota mimicking normal human gut microbiota reduces diabetes (Wen et al., 2008).

In the model of experimental autoimmune encephalomyelitis (EAE), oral administration of *Lactobacillus* was shown to attenuate disease (Maassen and Claassen, 2008) and cause changes in gut microbiota, decrease in proinflammatory cytokines, and increase in production of anti-inflammatory cytokines IL-10 and IL-13 (Ochoa-Repáraz et al., 2009). Similarly, oral administration of a zwitterionic capsular polysaccharide A from human commensal bacterium *B. fragilis* was found to protect against EAE through induction of oral tolerance mediated by tolerogenic CD103^+^ DCs and IL-10-producing FoxP3^+^ Tregs in the GALT (Ochoa-Repáraz et al., 2010). In addition, non-filamentous ATP-producing gut microbiota (Atarashi et al., 2008) and intestinal segmental filamentous bacteria (Ivanov et al., 2008) were reported to specifically prime the development of proinflammatory Th17-polarizing DCs but did not affect IFN-γ-producing Th1 cells and FoxP3^+^ Tregs subsets (Ivanov et al., 2009). Intestinal filamentous bacteria were found to contribute to the pathogenesis of autoimmune arthritis through stimulation of proinflammatory Th17 cells (Wu et al., 2010). Therefore, filamentous bacteria represent an intriguing example of commensal microbiota capable of shifting the mucosal effector T cell inflammatory/tolerogenic balance and thus affect the immune fitness of the individual (Ivanov and Littman, 2010).

### Conflict of Interest Statement

The authors declare that the research was conducted in the absence of any commercial or financial relationships that could be construed as a potential conflict of interest.
